# Aripiprazole monotherapy in patients with rapid-cycling bipolar I disorder: an analysis from a long-term, double-blind, placebo-controlled study

**DOI:** 10.1111/j.1742-1241.2008.01735.x

**Published:** 2008-05-01

**Authors:** D J Muzina, C Momah, J M Eudicone, A Pikalov, R D McQuade, R N Marcus, R Sanchez, B X Carlson

**Affiliations:** 1 Cleveland Clinic Lerner College of Medicine Cleveland, OH, USA; 2 Bristol-Myers Squibb, Plainsboro NJ, USA; 3 Otsuka America Pharmaceutical Inc. Rockville, MD, USA; 4 Otsuka Pharmaceutical Development & Commercialization, Inc. Princeton, NJ, USA; 5 Bristol-Myers Squibb Wallingford, CT, USA; 6 Bristol-Myers Squibb, Reuil-Malmaison Cedex Paris, France

## Abstract

**Aims:**

Rapid-cycling bipolar disorder is difficult to treat and associated with greater morbidity than non-rapid-cycling disease. This *post hoc* analysis evaluated 28 patients with rapid-cycling bipolar I disorder from a 100-week, double-blind, placebo-controlled study assessing long-term efficacy, safety and tolerability of aripiprazole in patients with bipolar I disorder (most recently manic/mixed).

**Methods:**

Following ≥ 6 consecutive weeks’ stabilisation with open-label aripiprazole, patients were randomised (1 : 1) to aripiprazole or placebo. Patients completing 26 weeks treatment without relapse could continue for a further 74 weeks. Primary end-point was time to relapse for manic, mixed or depressive symptoms, defined as discontinuation due to lack of efficacy. Safety assessments included adverse event (AE) monitoring and changes in weight and lipid, glucose and prolactin levels.

**Results:**

Of the 28 patients (aripiprazole, *n* = 14; placebo, *n* = 14) with rapid-cycling bipolar disorder, 12 (aripiprazole, *n* = 7; placebo, *n* = 5) completed the initial 26-week treatment period and three (all aripiprazole treated) completed the 100-week, double-blind period. Time to relapse was significantly longer with aripiprazole vs. placebo at week 26 [log-rank p = 0.033; 26-week hazard ratio = 0.21 (95% CI: 0.04, 1.03)] and week 100 [log-rank p = 0.017; 100-week hazard ratio = 0.18 (95% CI: 0.04, 0.88)]. The most commonly reported AEs with aripiprazole during the 100 weeks (≥ 10% incidence and twice placebo) were anxiety (*n* = 4), sinusitis (*n* = 4), depression (*n* = 3) and upper respiratory infection (*n* = 3). One aripiprazole-treated patient discontinued due to an AE (akathisia). There were no significant between-group differences in mean changes in weight or metabolic parameters.

**Conclusion:**

In this small, *post hoc* subanalysis, aripiprazole maintained efficacy and was generally well tolerated in the long-term treatment of rapid-cycling bipolar disorder. Further research with prospectively designed and adequately powered trials is warranted.

**What's known**

- Rapid cycling is a form of difficult to treat and at times refractory bipolar disorder with depression as a hallmark, and represents an important unmet need in bipolar disorder treatment.
- Few data are available regarding treatment, particularly in the long-term (> 26 weeks); this is especially pertinent in monitoring relapse prevention in patients with this form of bipolar disorder.
- This study is the first to report the long-term effects of aripiprazole in patients with rapid cycling.

**What's new**

- This *post hoc* analysis looked specifically at treatment of patients with rapid-cycling bipolar I disorder included in a 100-week, double-blind, placebo-controlled study to assess the long-term efficacy, safety and tolerability of aripiprazole. Despite the small sample size, encouraging results with aripiprazole (aripiprazole significantly delayed time to relapse vs. placebo) provide pilot data that calls for larger, double-blind, placebo-controlled investigation of aripiprazole's efficacy and safety in the treatment of rapid-cycling bipolar disorder.

## Introduction

Rapid-cycling bipolar disorder represents a phase or form of difficult to treat and at times refractory bipolar disorder with depression as its hallmark. Rapid cycling is an independent predictor of inadequate treatment response in patients with bipolar disorder (1–3) and is associated with greater morbidity vs. non-rapid-cycling disease (4). Rapid-cycling bipolar disorder is not well understood and also may be missed, thus representing an important unmet need in the treatment of bipolar I disorder (1,4,5).

The consequences of misdiagnosis in rapid-cycling bipolar disorder may be even greater than in non-rapid-cycling patients given the nature of this pernicious variant or phase of bipolar disorder (6). Indeed, misdiagnosis of rapid-cycling bipolar disorder has been associated with a twofold increase in the rate of hospitalisation compared with patients who were never diagnosed (7). Lifetime history of suicide attempts was also significantly elevated in patients with rapid-cycling bipolar disorder who had previously been misdiagnosed and whose clinical situation was complicated by substance use disorders, highlighting the importance of early and accurate diagnosis of rapid-cycling bipolar disorder to offer timely therapeutic interventions (7).

To be diagnosed with the Diagnostic and Statistical Manual of Mental Disorders, Fourth Edition (DSM-IV) course specifier of rapid cycling, patients must have at least four mood episodes within 12 months. These episodes must be demarcated by either a full or partial remission of at least 2 months’ duration or a switch to an episode of opposite polarity. In the Systematic Treatment Enhancement Program for Bipolar Disorder, a naturalistic study, ∼20% of patients were diagnosed with rapid-cycling bipolar disorder at study entry (8).

Controversies exist, however, regarding the necessary criteria for the diagnosis (particularly duration of episodes), and the aetiology of rapid-cycling bipolar disorder is poorly understood. Additionally, patients with rapid-cycling bipolar disorder are often excluded from clinical trials, so this lack of a full evidence base is associated with little consensus on the most appropriate treatment for these patients (5). Previous studies with lithium, divalproex and carbamazepine have indicated moderate-to-marked effects on mania, but poor-to-moderate effects in the treatment of the depressed phase of rapid-cycling bipolar disorder (5). In a prospective study with lamotrigine in which 182 patients with rapid-cycling bipolar disorder were randomised to lamotrigine (*n* = 93) or placebo (*n* = 89), there was no difference between treatment groups in the time to additional pharmacotherapy (primary end-point) (9). However, survival in the study (time to any premature discontinuation) was significantly longer with lamotrigine (p = 0.036) and a greater percentage of lamotrigine-treated patients were stable without relapse for 6 months of monotherapy vs. placebo-treated patients (41% vs. 26%; p = 0.03) (9).

Attention is shifting towards the use of atypical antipsychotics for the treatment of rapid-cycling bipolar disorder, albeit via analyses of data from acute bipolar mania or bipolar depression studies (10–14), open-label studies with atypical antipsychotics as adjunctive therapy (15–18) or case studies (19). Relatively fewer data exist regarding longer-term, maintenance therapy of rapid-cycling bipolar disorder.

Aripiprazole is pharmacologically distinct from other atypical antipsychotics, as the efficacy of this agent is mediated through its dopamine partial agonist activity at D_2_ and D_3_ receptors (20,21), 5-HT_2A_ antagonist activity (22,23) and 5-HT_1A_ partial agonist activity (23,24). The safety and efficacy of aripiprazole have been evaluated in both short- and longer-term studies in patients with bipolar I mania (25–27).

A longer-term, double-blind, placebo-controlled, 26-week study showed that aripiprazole was superior to placebo in delaying the time to relapse (28). Furthermore, in the 74-week, double-blind extension of that 26-week study (providing a total of 100 weeks’ double-blind treatment), time to relapse for the overall population continued to be significantly longer with aripiprazole vs. placebo, and aripiprazole maintained a good safety and tolerability profile (29).

Here, we present data from a *post hoc* analysis of patients with rapid-cycling bipolar I disorder who were included in that 100-week, double-blind, placebo-controlled study to assess the long-term efficacy, safety and tolerability of aripiprazole in these patients (28,29).

## Methods

### Study design

Details of the study methods have been described previously (28). Briefly, the original study was randomised, double-blind, parallel-group and placebo-controlled. During the initial stabilisation phase, patients were treated with open-label aripiprazole (15 or 30 mg/day). Patients who achieved a Young Mania Rating Scale (YMRS) total score ≤ 10 and a Montgomery–Åsberg Depression Rating Scale (MADRS) total score ≤ 13 over a minimum of six consecutive weeks were eligible for double-blind treatment.

In the 100-week, prospective double-blind period, patients were randomised (1 : 1) to receive either aripiprazole or placebo for an initial 26-week treatment period, the results of which have been reported elsewhere (28). Patients who completed the 26-week period without relapse were offered to continue a further 74 weeks of double-blind treatment under the same regimen. Results from the 100-week treatment period have been reported elsewhere (29). Entrance into the 74-week double-blind, extension phase ended when 45 patients had relapsed. All patients continued in a double-blind, placebo-controlled fashion in the study until the last randomised patient completed the 26-week phase. At that time, the study was terminated, and any patient remaining in the 74-week phase was discontinued, regardless of the time point they had reached.

Study visits took place at randomisation (day 1 of the double-blind phase), weekly for weeks 1–4, biweekly for weeks 6–28, monthly for weeks 28–52 and bimonthly for weeks 52–100. All study sites received prior Institutional Review Board (IRB)/Institutional Ethics Committee (IEC) approval before study initiation.

### Patients

All patients provided written informed consent, as required by the IRB/IEC. Participants met the criteria for bipolar I disorder according to the DSM-IV. Diagnoses were performed using the Structured Clinical Interview for DSM or the Mini-International Neuropsychiatric Interview. The *post hoc* analyses reported here consider only those patients diagnosed with rapid-cycling bipolar I disorder.

Details of the inclusion and exclusion criteria are available elsewhere (28). Briefly, patients were eligible for entry into the stabilisation phase if they had either recently completed a 3-week, placebo-controlled acute mania study of aripiprazole, if they met eligibility criteria for an acute mania study but had declined participation, or if they had experienced a manic or mixed episode requiring hospitalisation and treatment within the previous 3 months. All psychotropic medications, except lorazepam and anticholinergic agents, were discontinued prior to enrolment.

### Dosing schedule

Study medication was administered orally, once daily, at approximately the same time each day. During the stabilisation phase, patients received open-label aripiprazole, initially 30 mg/day. Dose decrease to 15 mg/day was permitted at any time, depending on tolerability. Following entry to the double-blind phase, patients were assigned, in a double-blind manner, to continue the dose of aripiprazole they were taking at the end of stabilisation or to receive placebo. Based on the investigator's assessment of therapeutic effect and tolerability, the aripiprazole dose could be increased to 30 mg/day or decreased to 15 mg/day at any time.

### Efficacy measures

The primary efficacy end-point was the time to relapse for a mood episode (manic, depressive or mixed) during the initial 26-week, double-blind period of the study. Relapse was defined as discontinuation because of lack of efficacy (indicated by hospital admission because of a mood episode and/or addition to, or increase in, psychotropic medication other than study drug for manic and/or depressive symptoms). Additional efficacy measures included the mean change from double-blind randomisation to end-point in YMRS and MADRS total scores, and time to relapse over the course of the additional 74-week, double-blind extension phase.

### Safety measures

Treatment-emergent adverse events (AEs; using COSTART terminology) were collected from study commencement. Vital sign measurements and changes in body weight were also assessed. Clinical laboratory tests included fasting measurements of triglyceride, high-density lipoprotein cholesterol, low-density lipoprotein cholesterol, total cholesterol, glucose and prolactin levels.

### Data analysis

The primary efficacy measure – time to relapse – was evaluated using statistical methods designed for the time-to-event data. Kaplan–Meier survival curves were generated for the time-to-relapse data, and between-group differences were tested using log-rank tests at an alpha level of 0.05 to indicate statistical significance. Patients who did not relapse, including those who discontinued participation early for reasons other than relapse, were censored on the date of their last efficacy evaluation or the last dose of study medication.

Treatment differences for the incidence of clinically significant weight gain were evaluated using Fisher's exact test. Analysis of covariance (ANCOVA) modelling was used to analyse change from baseline to last observation carried forward (LOCF) end-point in laboratory parameters. ANCOVA models included the baseline measure as covariate and treatment group as main effect.

Primary presentations of results from ANCOVA and analysis of variance were the model-based estimates corresponding p-values for the test of treatment differences. Changes from baseline were derived by subtracting the baseline score from that of the LOCF end-point.

Analyses of laboratory parameters were performed on LOCF data for the 26-week maintenance phase and the combined 26-week maintenance and 74-week double-blind, extension phases. LOCF values at week 26 for those patients who did not enter the 74-week double-blind, extension phase were carried forward to week 100.

All safety and efficacy analyses were performed using sas statistical software, version 6.12 or higher (SAS Institute Inc., Cary, NC). For the comparison of aripiprazole with placebo, two-tailed tests were used to determine statistical significance and p *≤* 0.05 was considered to be statistically significant.

## Results

### Patient disposition and characteristics

Detailed presentations of patient disposition and demographics in the total population during stabilisation and the initial 26-week double-blind phase of the study (28) and the entire 100-week double-blind phase (29) are provided elsewhere.

Of the 161 patients in the total population, 28 had a history of rapid-cycling bipolar I disorder, met the stabilisation criteria and were randomised to aripiprazole (*n* = 14) or placebo (*n* = 14). Of these, 12 patients [placebo, 5/14 (36%); aripiprazole, 7/14 (50%)] completed the initial 26-week, double-blind period, and entered the 74-week, double-blind extension phase; three patients completed the full 100-week, double-blind period [placebo, 0/14 (0%) patients; aripiprazole, 3/14 (21%) patients] (Table 1). The baseline demographics and characteristics in the subgroup of patients with rapid-cycling bipolar disorder are shown in Table 2.

**Table 1:** Disposition of patients with rapid-cycling bipolar disorder

	Placebo, *n* (%)	Aripiprazole, *n* (%)
**26-week, double-blind phase**		
Randomised	14 (100)	14 (100)
Discontinued	9 (64)	7 (50)
Lack of efficacy	7 (50)	2 (14)
Adverse event	0	1 (7)
Consent withdrawal	0	2 (14)
Lost to follow-up	1 (7)	0
Other	1 (7)	2 (14)
Completed	5 (36)	7 (50)
**74-week, double-blind extension phase**		
Entered	5 (36)	7 (50)
Discontinued	5 (36)	4 (29)
Lack of efficacy	1 (7)	0
Adverse event	0	0
Consent withdrawal	0	3 (21)
Lost to follow-up	0	0
Other	4 (29)	1 (7)
Completed	0	3 (21)

**Table 2:** Baseline demographics and characteristics

	Placebo (*n* = 14)	Aripiprazole (*n* = 14)
Mean ± SD age (years)	38.8 ± 11.8	37.6 ± 12.5
**Gender, *n* (%)**		
Male	4 (28.6)	5 (35.7)
Female	10 (71.4)	9 (64.3)
**Race, *n* (%)**		
White	12 (85.7)	9 (64.3)
Hispanic	2 (14.3)	3 (21.4)
Other	0	2 (14.3)
Mean ± SD body weight	88.7 ± 14.9	95.5 ± 18.5
Mean ± SD YMRS total score	2.0 ± 2.1	3.6 ± 3.1
Mean ± SD MADRS total score	3.5 ± 3.1	4.7 ± 4.0

SD, standard deviation; YMRS, Young Mania Rating Scale; MADRS, Montgomery–Åsberg Depression Rating Scale.

### Medications

The mean ± standard deviation aripiprazole dose during the last 7 days of the initial 26-week double-blind phase was 25.3 ± 7.2 mg (*n* = 14), similar to that during the last 7 days of the 100-week study (23.6 ± 8.0 mg; *n* = 7).

### Efficacy

**Figure 1:**
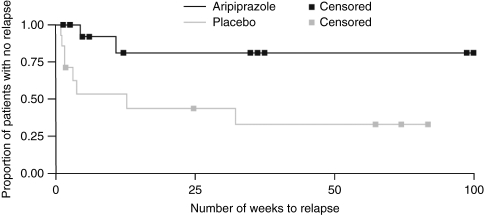
Time to relapse during the 100-week double-blind treatment with aripiprazole or placebo. Numbers of subjects remaining at each censoring time point are indicated

As observed in the original study in the total population of both rapid-cycling and non-rapid-cycling patients (28,29), time to relapse was significantly longer with aripiprazole vs. placebo treatment at both 26 weeks [log-rank p = 0.033; 26-week hazard ratio = 0.21 (95% CI: 0.04, 1.03)] and 100 weeks [log-rank p = 0.017; 100-week hazard ratio = 0.18 (95% CI: 0.04, 0.88)] in patients with rapid-cycling bipolar disorder (Figure 1).

The median survival time in the placebo group was 118 days (95% CI: 14, not evaluable) at which time approximately 45% of subjects with rapid-cycling bipolar disorder had not yet relapsed. The median survival time for the aripiprazole group was not evaluable. At the time of the last relapse event in the study period, which occurred at day 101, 81% of aripiprazole-treated subjects with rapid-cycling bipolar disorder had not yet relapsed. In the subpopulation of non-rapid-cycling patients, median time to survival in the placebo group was 203 days (95% CI: 128, 661), while the median time to survival in the aripiprazole group was not evaluable.

The YMRS total scores increased in both groups. The mean ± standard error (SE) increase in YMRS total score (LOCF) was numerically smaller with aripiprazole vs. placebo from baseline at week 26 (+3.0 ± 2.0 vs. +6.6 ± 2.0; p = 0.213; effect size 0.506) and week 100 (+2.6 ± 2.6 vs. +9.5 ± 2.6; p = 0.077; effect size 0.730). These results in patients with rapid-cycling bipolar disorder were consistent with those in the original study in the total population of patients (28,29). In the subpopulation of non-rapid-cycling patients, the mean ± SE increase in YMRS total scores from baseline of the double-blind phase to week 100 (LOCF) was significantly smaller with aripiprazole vs. placebo (+5.3 ± 1.4 vs. +9.4 ± 1.4; p = 0.0374; effect size 0.369).

As observed in the original study in the total population of patients (28,29), the MADRS total scores increased in both treatment groups in the subpopulation of rapid-cycling patients, with no statistically significant difference with aripiprazole vs. placebo in the mean change in MADRS total score (LOCF) from baseline at week 26 (+8.3 ± 3.3 vs. +11.5 ± 3.3; p = 0.519; effect size 0.251) or week 100 (+7.7 ± 3.3 vs. +12.5 ± 3.3; p = 0.304; effect size 0.403). In non-rapid-cycling patients, the change in MADRS total score from baseline of the double-blind phase to week 100 (LOCF) was not significantly different between aripiprazole and placebo (+5.8 ± 1.4 vs. +7.0 ± 1.3; p = 0.5499; effect size 0.106).

### Safety

In the 26-week, double-blind treatment phase, treatment-emergent AEs reported in the aripiprazole group were: anxiety (*n* = 4), depression (*n* = 3), emotional lability (*n* = 2), insomnia (*n* = 2), nervousness (*n* = 2), tremor (*n* = 2), asthenia (*n* = 2), headache (*n* = 2), extremity pain (*n* = 2), neck rigidity (*n* = 2) and dental disorder (*n* = 2). Treatment-emergent AEs in the placebo group were: headache (*n* = 1), anxiety (*n* = 1) and depression (*n* = 1).

Treatment-emergent AEs at week 100 are shown in Table 3. Akathisia, sinusitis, upper respiratory infection, diarrhoea, pharyngitis, acne, urinary tract infection, flu syndrome, infection and dry mouth were additional treatment-emergent AEs that occurred as a result of treatment with aripiprazole during the 74-week extension phase of the study (Table 3).

**Table 3:** Treatment-emergent adverse events with an incidence of ≥ 10% in either treatment group during the 100-week study

Adverse event, *n* (%)	Placebo (*n* = 14)	Aripiprazole (*n* = 14)
**During 26-week, double-blind phase**		
Anxiety	1 (7.1)	4 (28.6)
Depression	1 (7.1)	3 (21.4)
Headache	1 (7.1)	2 (14.3)
Asthenia	0	2 (14.3)
Extremity pain	0	2 (14.3)
Neck rigidity	0	2 (14.3)
Insomnia	0	2 (14.3)
Tremor	0	2 (14.3)
Emotional lability	0	2 (14.3)
Nervousness	0	2 (14.3)
Dental disorder	0	2 (14.3)
**During 74-week, double-blind extension phase**		
Upper respiratory infection	1 (7.1)	3 (21.4)
Sinusitis	0	4 (28.6)
Infection	1 (7.1)	2 (14.3)
Akathisia	1 (7.1)	2 (14.3)
Insomnia	2 (14.3)	0
Urinary tract infection	0	2 (14.3)
Pharyngitis	0	2 (14.3)
Flu syndrome	0	2 (14.3)
Diarrhoea	0	2 (14.3)
Dry mouth	0	2 (14.3)
Acne	0	2 (14.3)

One patient in the aripiprazole group discontinued because of an AE (akathisia) during the initial 26-week, double-blind phase. No other discontinuations because of an AE occurred during the remainder of the 100-week study in either the placebo or aripiprazole groups.

Mean ± SE weight change from baseline at week 26 was −3.8 ± 3.4 kg with aripiprazole (*n* = 11) and +0.3 ± 4.1 kg with placebo (*n* = 9) (p = 0.444; LOCF). At week 100, mean ± SE weight change from baseline was −4.6 ± 2.7 kg with aripiprazole (*n* = 11) and +0.8 ± 3.4 kg with placebo (*n* = 7) (p = 0.230; LOCF).

At week 26, none of the seven patients evaluated in the placebo group experienced clinically significant weight increase (≥ 7%) compared with one of 10 patients in the aripiprazole group (p = 0.588; LOCF). During the 74-week extension phase, one patient in the placebo group and one in the aripiprazole group experienced clinically significant weight gain. In total at week 100, one of seven placebo-treated patients and two of 11 aripiprazole-treated patients experienced clinically significant weight gain (p = 0.472; LOCF).

At week 100, there were no statistically significant changes from baseline in glucose, lipid or prolactin levels with either aripiprazole or placebo (p > 0.05) (Table 4). Furthermore, there were no statistically significant differences between aripiprazole and placebo across these metabolic parameters.

**Table 4:** Mean changes in fasting clinical laboratory measures from baseline at week 100 (last observation carried forward; safety sample)

	Placebo	Aripiprazole*
**Total cholesterol (mg/dl)**		
*N*	7	8
Days on treatment, median (range)	302 (14–624)	281 (12–700)
Baseline (mean ± SD)	202 ± 44	172 ± 16
Week 100 (mean change ± SE)	+11 ± 10	+3 ± 9
**HDL-cholesterol (mg/dl)**		
*N*	7	8
Days on treatment, median (range)	302 (14–624)	281 (12–700)
Baseline (mean ± SD)	52 ± 16	47 ± 7
Week 100 (mean change ± SE)	+4.2 ± 2.7	−0.04 ± 2.5
**LDL-cholesterol (mg/dl)**		
*N*	7	8
Days on treatment, median (range)	302 (14–624)	281 (12–700)
Baseline (mean ± SD)	117 ± 38	100 ± 16
Week 100 (mean change ± SE)	+14 ± 10	+8 ± 9
**Triglycerides (mg/dl)**		
*N*	4	6
Days on treatment, median (range)	419 (118–579)	281 (56–700)
Baseline (mean ± SD)	199 ± 90	93 ± 24
Week 100 (mean change ± SE)	−55 ± 30	+29 ± 23
**Serum glucose (mg/dl)**		
*N*	4	6
Days on treatment, median (range)	419 (118–579)	281 (56–700)
Baseline (mean ± SD)	85 ± 7	91 ± 13
Week 100 (mean change ± SE)	+4.8 ± 7.8	+3.5 ± 6.3
**Prolactin (ng/ml)**		
*N*	11	12
Days on treatment, median (range)	42 (14–624)	275 (12–700)
Baseline (mean ± SD)	11 ± 9	13 ± 8
Week 100 (mean change ± SE)	−1.6 ± 1.7	+1.3 ± 1.6

* All values p > 0.05 vs. placebo. SD, standard deviation; SE, standard error; HDL, high-density lipoprotein; LDL, low-density lipoprotein.

## Discussion

In this *post hoc* analysis, aripiprazole significantly delayed the time to relapse in patients with rapid-cycling bipolar I disorder (most recently manic/mixed) from a 100-week, double-blind, placebo-controlled study.

Of the rapid-cycling patients who were initially stabilised on aripiprazole for six consecutive weeks and then randomised to aripiprazole, 3/14 patients completed the 100-week trial, whereas 0/14 placebo-treated patients completed the trial. In comparison, in the original study in the total population of patients with bipolar disorder, 5/83 placebo-treated and 7/78 aripiprazole-treated patients completed the 100-week trial (29). This high attrition rate is not uncommon in long-term trials (13,30), but this can limit the usefulness of long-term results, and highlights the difficulties inherent in treating bipolar patients.

The changes in YMRS and MADRS total scores from baseline in the aripiprazole group in this analysis of patients with rapid-cycling bipolar disorder were similar to the changes observed in the overall population of aripiprazole-treated patients with bipolar I disorder in the original study (28,29) and in the subpopulation of patients with non-rapid-cycling bipolar disorder. With aripiprazole monotherapy, small increases in YMRS and MADRS scores were observed over the course of the 100-week study, yet the average total scores remained below the stability criteria used in the open-label phase (i.e., YMRS total ≤ 10 and MADRS total ≤ 13). Thus, aripiprazole monotherapy appears to have a consistent efficacy profile in maintaining mood stability in patients with bipolar I disorder, including those with rapid-cycling bipolar disorder.

Long-term aripiprazole monotherapy was generally well tolerated in this population of rapid-cycling patients, with the majority of treatment-emergent AEs occurring during the 26-week, double-blind treatment phase of the study. The effect of aripiprazole on body weight in this study is of interest as obesity is more prevalent in patients with bipolar disorder than in the general population (31,32) and has been noted as a correlate of poor outcome in these patients (33). Importantly, in the current study, aripiprazole was not associated with either significant weight gain or clinically relevant changes in metabolic parameters, including glucose, lipid or prolactin levels, during long-term treatment of patients with rapid-cycling bipolar disorder.

Much insight into the safety and efficacy of atypical antipsychotics for the treatment of rapid-cycling bipolar disorder is derived from subanalyses of larger studies in patients with bipolar disorder. Such analyses have indicated the short-term efficacy of quetiapine for the treatment of depressive symptoms (14) and of olanzapine for the treatment of manic symptoms (10) in rapid-cycling patients. However, treatment response in rapid-cycling patients should be assessed over at least 4 months or three cycle lengths, as resolution of an acute cycle is not necessarily evidence that a treatment has worked (5). A previous *post hoc* analysis of a 47-week, randomised, double-blind study comparing olanzapine (5–20 mg/day) with divalproex sodium (500–2500 mg/day) in 251 patients with bipolar mania (manic or mixed) showed greater overall improvement with non-rapid-cycling vs. rapid-cycling patients during long-term treatment (12), with little difference between olanzapine and divalproex in the improvement in mania (12). Most pertinent to the results of the current study, an analysis of 179 patients with rapid-cycling bipolar disorder from a double-blind, placebo-controlled study of the safety and efficacy of olanzapine monotherapy in relapse prevention in patients with bipolar I disorder (manic or mixed) indicated a longer time to relapse with olanzapine vs. placebo during up to 48-weeks maintenance therapy (13). One prospective, randomised study investigated lamotrigine monotherapy in patients with rapid-cycling bipolar disorder (9). In that study, there was no difference with lamotrigine vs. placebo in the time to additional pharmacotherapy (primary end-point) (9), but more patients were stable without relapse for 6 months with lamotrigine vs. placebo (9). Of note, the 100-week study described in the current analysis of rapid-cycling patients is the longest double-blind, placebo-controlled study to date investigating the efficacy, tolerability and safety of any agent other than lithium for the prevention of relapse in the treatment of bipolar I disorder.

Interpretation of the current study is limited by the *post hoc* nature of the analysis and by the small population size (*n* = 28); however, the paucity of other long-term data in patients with rapid-cycling bipolar disorder should be acknowledged. It should also be noted that as all patients were stabilised for at least 6 weeks on aripiprazole prior to randomisation, the study represents an enriched population who continued into the double-blind treatment. Thus, the results are only applicable to those rapid-cycling patients who respond to and are stabilised on aripiprazole following a manic or mixed episode. The study focused on patients with an index episode of bipolar mania, and further studies would be required to more specifically determine the efficacy of aripiprazole in the prophylaxis of depressive episodes in rapid-cycling bipolar disorder. This would be of particular relevance, as patients with rapid-cycling bipolar disorder present more frequently in the depressed phase than the manic phase (6) and have substantial depressive morbidity compared with bipolar patients without rapid cycling (34).

These encouraging results with up to 100 weeks of aripiprazole treatment call for further research with more prospectively designed and adequately powered trials to better investigate the efficacy and safety of long-term treatment of rapid-cycling bipolar disorder. Maintenance studies that stratify patients according to index mood polarity at study entry (35) and protocols to assess the benefits of combination regimens (5) using atypical antipsychotics such as aripiprazole would be of substantial clinical interest.
